# Variations in BDNF and Their Role in the Neurotrophic Antidepressant Mechanisms of Ketamine and Esketamine: A Review

**DOI:** 10.3390/ijms252313098

**Published:** 2024-12-05

**Authors:** Simone Pardossi, Andrea Fagiolini, Alessandro Cuomo

**Affiliations:** Department of Molecular Medicine, University of Siena School of Medicine, 53100 Siena, Italy; s.pardossi@student.unisi.it (S.P.); alessandro.cuomo@unisi.it (A.C.)

**Keywords:** BDNF, ketamine, esketamine, major depressive disorder, treatment-resistant depression

## Abstract

Brain-derived neurotrophic factor (BDNF) is critical for neuroplasticity, synaptic transmission, and neuronal survival. Studies have implicated it in the pathophysiology of depression, as its expression is significantly reduced in brain areas such as the prefrontal cortex and hippocampus in patients with depression. Our narrative review focuses on the relationship between BDNF, ketamine, and esketamine, specifically by summarizing human studies investigating BDNF variations in patients treated with these two drugs. BDNF plays a pivotal role in neuroplasticity and neurotrophic mechanisms that can be enhanced by traditional antidepressants, which have been shown to increase BDNF levels both peripherally and in targeted brain regions. Ketamine and its S-enantiomer, esketamine, exert both rapid and sustained antidepressant effects through activation of glutamate-related pathways, with neurotrophic effects involving BDNF, as demonstrated in experimental studies. However, clinical findings have shown mixed results, with most indicating an increase in plasma BDNF in patients treated with intravenous ketamine, although some studies contradict these findings. In addition to this, there are few studies of BDNF and esketamine. Currently, the limited number of studies suggests the need for further research, including larger sample sizes and investigations of BDNF and intranasal esketamine, which has been approved by several regulatory agencies for the treatment of treatment-resistant depression.

## 1. Introduction

The pathophysiology of major depressive disorder (MDD) remains incompletely understood, and several hypotheses have been proposed to explain its complex and heterogeneous nature [1]. Among these, the neurotrophic hypothesis has received considerable attention, focusing in particular on the role of brain-derived neurotrophic factor (BDNF), a protein essential for neuroplasticity, synaptic transmission, and neuronal survival [2,3]. Alterations in BDNF levels have been implicated in the pathogenesis of MDD, with evidence suggesting that reduced BDNF expression in key brain regions, such as the hippocampus and prefrontal cortex, may contribute to the development of depressive symptoms [4,5]. The neurotrophic hypothesis proposes that downregulation of BDNF and subsequent impairment of neuroplasticity are central to the pathophysiology of MDD, linking the disorder to broader mechanisms of neuronal atrophy and synaptic loss [6]. Conventional antidepressants, including selective serotonin reuptake inhibitors (SSRIs), have been shown to gradually increase BDNF levels with chronic treatment, which correlates with their delayed therapeutic onset [2,7].

However, this slow onset of action poses significant challenges for patients, particularly those with severe or treatment-resistant depression, for whom a rapid therapeutic response is critical. Understanding the mechanisms of depression is crucial for developing effective treatments. Approximately 30% of patients fail to respond adequately to first-line therapies, including SSRIs or serotonin-norepinephrine reuptake inhibitors (SNRIs). This phenomenon, known as treatment-resistant depression (TRD), is defined as a failure to achieve symptom remission despite adequate trials of at least two antidepressants from different pharmacological classes [8,9]. TRD represents a significant clinical challenge, as it is associated with poor prognoses, including higher rates of chronicity, comorbid anxiety disorders, cognitive decline, and suicide risk [10].

Studies have demonstrated that with each unsuccessful treatment attempt, the likelihood of achieving remission diminishes [11]. For instance, the Sequenced Treatment Alternatives to Relieve Depression (STAR*D) trial reported a cumulative remission rate of 67% after up to four antidepressant treatment trials. However, when adhering strictly to protocol-specified criteria, the remission rate was approximately 35% [11].

Esketamine, the S-enantiomer of ketamine, has revolutionized the treatment landscape for depression by providing rapid antidepressant effects. It has been approved by regulatory agencies, including the U.S. Food and Drug Administration (FDA), for the treatment of TRD [12]. Unlike traditional antidepressants, which often take weeks to show efficacy, esketamine has demonstrated the ability to produce significant symptom relief rapidly, with substantial reductions in depressive symptoms [13,14]. A 2021 review analyzing 64 randomized controlled trials with 5299 participants showed that both ketamine and esketamine might increase response and remission rates within 24 h compared to placebo [15]. Recent evidence also suggests long-term efficacy [16]. This rapid antidepressant effect is thought to involve modulation of glutamatergic transmission, leading to activation of downstream pathways that enhance synaptic plasticity [13]. In addition, the racemic mixture of S-ketamine and R-ketamine, commonly referred to simply as ketamine, has also shown similar rapid antidepressant effects [17]. A key mechanism of ketamine and esketamine may be the upregulation of BDNF levels [18,19], which plays a critical role in synaptic resilience and neuroplasticity [20].

The aim of this narrative review is to first present the current knowledge in the literature regarding BDNF, its association with depression, and its relationship with traditional antidepressant treatments. We will then focus on the relationship between BDNF, ketamine, esketamine, and their antidepressant effects. This will include an examination of the known mechanisms of ketamine through experimental studies, as well as a discussion of human studies that have examined BDNF variations in patients treated with ketamine and esketamine. To achieve this, we conducted a comprehensive literature search using databases such as PubMed, Scopus, and Google Scholar, with keywords including ‘ketamine’, ‘esketamine’, ‘BDNF’, ‘neurotrophism’, and ‘neuroplasticity’. We carefully examined the articles retrieved, focusing on studies published in English, and extracted the necessary information to introduce the topic. For the molecular mechanisms underlying the link between ketamine, esketamine, and BDNF, we considered preclinical studies. We then focused on human studies in the literature to better understand the impact of ketamine and esketamine on BDNF levels in patients.

The aim of our work is to elucidate the current knowledge on the impact of ketamine and esketamine on BDNF, thereby exploring one of the key mechanisms underlying their action, which contributes to the innovative efficacy of these novel antidepressant treatments.

## 2. BDNF: Functions, Distribution, and Implications

BDNF is a critical neurotrophic factor that contributes significantly to the development, maintenance, and plasticity of neurons in the central nervous system [21]. BDNF is particularly known for its role in promoting neurogenesis, enhancing synaptic plasticity, and supporting neuronal survival [21]. It is produced as a precursor protein known as proBDNF, which is subsequently cleaved into its mature, biologically active form. This cleavage can occur intracellularly, within the trans-Golgi network by enzymes such as furin, or extracellularly, after the release of proBDNF into the synaptic cleft, by matrix metalloproteinases or the tissue plasminogen activator/plasmin system [22,23]. Mature BDNF and its precursor pro-BDNF trigger distinct intracellular signaling pathways [22,23]. Pro-BDNF binds to the low-affinity neurotrophin receptor p75: this interaction can activate signaling cascades involving c-Jun N-terminal kinase (JNK), Ras homolog gene family member A (RhoA), and nuclear factor kappa B (NF-κB), which are associated with processes like growth cone retraction and apoptosis [22,23,24]. In contrast, mature BDNF interacts with the high-affinity tropomyosin receptor kinase B (TrkB) receptor and other signaling pathways [22]. When mature BDNF binds to TrkB, it causes the receptor to dimerize and autophosphorylate its tyrosine kinase, leading to the initiation of intracellular signaling pathways and the enhancement of N-methyl-D-aspartate (NMDA) receptor activity [25]. The BDNF-TrkB interaction activates three major signaling pathways [22]: the phospholipase Cγ (PLCγ) pathway, which activates protein kinase C (PKC); the phosphatidylinositol 3-kinase (PI3K) pathway, which triggers the AKT kinase; and the mitogen-activated protein kinase (MAPK) pathway, which affects several downstream targets. These pathways allow BDNF to exert a wide range of effects, from rapid synaptic modulation via PLCγ-induced calcium release to more sustained changes in gene expression via the PI3K and MAPK pathways [22,26].

BDNF is predominantly expressed in regions such as the hippocampus, amygdala, cerebellum, and cortex [27], which are critical for learning, memory, and emotional regulation [28]. Studies confirm that BDNF mRNA and protein levels are particularly high in the hippocampus, where it is involved in synaptic plasticity and memory formation [21,22]. In addition, BDNF expression is highly activity-dependent, with neuronal activity significantly influencing its synthesis and release [29].

Fluctuations in BDNF levels are closely associated with several neurological and psychiatric disorders. For example, reduced BDNF levels are associated with mood disorders [30] and neurodegenerative diseases such as Alzheimer’s disease [31]. In schizophrenia, altered BDNF levels have been implicated in impaired synaptic plasticity and cognitive dysfunction [32].

## 3. BDNF, Depression, and Antidepressants

BDNF plays a critical role in synapse formation, plasticity, and neuronal survival, making it an important focus in studies of MDD. Chronic stress, a well-established risk factor for MDD, has been shown to decrease BDNF expression in critical brain regions such as the hippocampus and prefrontal cortex (PFC), contributing to the neuronal atrophy observed in depression [2,22,33]. This finding supports the “neurotrophic hypothesis” of depression, which suggests that reduced BDNF levels in individuals with MDD are associated with impaired neurogenesis, particularly in regions involved in emotion and memory [3]. The decrease in BDNF is associated with reduced neuronal growth, poorer synapse formation, and increased cell death, all of which contribute to the development and persistence of depressive symptoms [3].

Interestingly, while chronic stress generally suppresses BDNF expression in the hippocampus and PFC, it can increase BDNF levels in other brain regions, such as the nucleus accumbens and amygdala, where this increased activity may influence the synaptic plasticity associated with depressive symptoms [2]. Elevated BDNF levels have been observed in the nucleus accumbens tissue of human patients with MDD [34], and neuroanatomical studies have also identified amygdala hypertrophy as another hallmark of MDD [35,36]. In addition, post-mortem studies have shown decreased levels of BDNF in the cerebral cortex of individuals with depression who died by suicide, along with decreased activation of downstream signaling pathways such as TrkB-ERK and Akt [37] and patients with depression also have altered levels of serum BDNF [38]. A 2022 study highlighted a significant reduction in BDNF plasma levels among patients with first-episode, unmedicated MDD compared to healthy controls [39]. Moreover, a recent systematic review has shown that, although the results of some of the reviewed studies are occasionally inconsistent, there is a general trend indicating that patients with MDD tend to have lower serum levels of BDNF compared to healthy individuals [40]. This finding reinforces the idea that BDNF could serve as a valuable biomarker in understanding the biological underpinnings of depression and monitoring treatment outcomes. Taken together, these findings suggest a complex role for BDNF signaling in MDD, with increased activity in the amygdala and nucleus accumbens and decreased activity in the hippocampus and prefrontal cortex [22].

Conversely, antidepressant treatment is associated with an increase in BDNF levels, suggesting a restorative effect on neural circuits impaired by depression [38]. This is consistent with meta-analytic data indicating that successful antidepressant treatment is often associated with normalization of BDNF levels, particularly in those who experience significant symptom relief [38]. A study that compiled evidence from several meta-analyses found a negative correlation between blood BDNF levels and symptom severity, while no association with suicidality was detected. Additionally, an increase in blood BDNF levels following antidepressant treatment was observed, proportional to the improvement in symptoms [3]. BDNF levels appear to rise in both treatment responders and remitters, while remaining stable in non-responders [3].

BDNF has emerged as a critical component in understanding the mechanism of action of antidepressants, particularly in the context of MDD. Antidepressants, particularly selective serotonin reuptake inhibitors (SSRIs) and serotonin-norepinephrine reuptake inhibitors (SNRIs), have been shown to increase BDNF levels both in peripheral blood and in specific brain regions such as the hippocampus, which is critical for mood regulation and neurogenesis [3,41]. Early research found that both antidepressants and electroconvulsive therapy (ECT) increase BDNF and TrkB mRNA levels in the hippocampus and FC, aligning with the timing of antidepressant effects [42,43,44].

It was also discovered that administering BDNF to the dentate gyrus and CA3 regions of the hippocampus produced antidepressant effects within a few days, primarily through TrkB activation and the MEK-ERK signaling pathway [45]. Several hypotheses have been proposed regarding the mechanisms by which BDNF mediates antidepressant effects: one key hypothesis is that BDNF modulates synaptic plasticity and neurogenesis, particularly in the hippocampus, where activation of TrkB receptors by BDNF is essential for remodeling neural circuits and improving mood and behavior [41]. Chronic activation of TrkB by antidepressants has been proposed to induce a juvenile-like state of plasticity in the adult brain, contributing to the therapeutic effects of these drugs [41,46]. In addition, antidepressants may promote BDNF expression not only in neurons but also in astrocytes and microglia, with therapeutic efficacy potentially mediated through BDNF expression in these cell types as well [46]. Another mechanism involves epigenetic regulation of the BDNF gene, where modifications such as DNA methylation and histone modification affect BDNF expression and consequently response to antidepressants [41]. In particular, Both DNA methylation and histone modifications are known to regulate BDNF transcription in response to environmental stimuli like stress, trauma, and drug exposure [47,48,49]. In MDD, DNA hypermethylation at specific BDNF promoters, particularly at promoter P4, is frequently observed [50]. This modification correlates with a significant reduction in BDNF expression, which has been documented both in post-mortem brain tissues and in the blood of individuals with depression, including those exhibiting suicidal behaviors [48]. Histone modifications further contribute to the complexity of BDNF regulation in depression: repressive histone marks like H3K27me3 have been found to accumulate in stress-sensitive brain regions, such as the hippocampus and nucleus accumbens, leading to decreased BDNF levels [47]. This downregulation of BDNF is closely associated with impaired synaptic plasticity, a hallmark of depression and mood dysregulation [47,48] Conversely, animals resilient to chronic stress exhibit an adaptive upregulation of BDNF, mediated by permissive histone modifications such as H3K4me3, which help maintain neural plasticity and mood stability [51]. One of the most studied BDNF polymorphisms in depression and depression treatment is the Val66Met variant (rs6265), which results in a substitution of valine (Val) with methionine (Met) at codon 66 in the proBDNF protein [52]. This polymorphism significantly affects BDNF secretion and activity by impairing its intracellular trafficking and activity-dependent release [52]. The Met allele has been associated with reduced hippocampal volume, altered synaptic plasticity, and diminished stress resilience, all of which are critical factors in the pathophysiology of depression [52]. Met allele carriers may influence the response to several antidepressants; however, research on the effects of this mutation remains inconclusive and sometimes contradictory [53,54,55].

## 4. Ketamine Mechanism of Action and BDNF Involvement

Ketamine exists as a racemic mixture of two isomers: S-ketamine (esketamine) and R-ketamine (arketamine): together, these two enantiomers contribute to the molecular effects outlined below.

Ketamine, a non-competitive antagonist of the NMDA receptor, has attracted considerable interest for its ability to produce rapid and sustained antidepressant effects, particularly in individuals with treatment-resistant depression [18]. The rapid onset of these effects, often evident within hours, and their persistence for weeks after a single dose suggest that ketamine’s action involves more complex processes than simple NMDA receptor blockade, particularly those related to synaptic plasticity and neurotrophic signaling, including BDNF pathways [18,19]. Ketamine was originally developed as an anesthetic, but its anesthetic and antidepressant effects are closely linked through its initial action as a non-competitive NMDA receptor antagonist [56]. At anesthetic doses, ketamine globally suppresses cortical and subcortical activity, resulting in dissociation and analgesia [57]. In contrast, at sub-anesthetic doses used for depression, ketamine preferentially blocks NMDA receptors on GABAergic interneurons [44,58]. This disinhibition of glutamatergic pyramidal neurons triggers a rapid surge in extracellular glutamate, with notable effects on synaptogenesis [44,58]. Taking a broader perspective on the effects of ketamine on synaptogenesis and neuroplasticity, particularly its long-term effects, one of its primary actions is its profound impact on the medial prefrontal cortex (mPFC) and hippocampus, areas that are integral to mood regulation [59]. In the mPFC, ketamine rapidly increases synaptic connectivity by increasing both the number and functionality of synapses on layer V pyramidal neurons, which are often compromised under chronic stress [44,60]. Ketamine has been shown to counteract the loss of dendritic spines caused by chronic unpredictable stress (CUS), restoring synaptic deficits and reinstating coordinated neural activity predictive of escape behavior, an indicator of antidepressant efficacy [61]. Notably, these synaptic changes occur rapidly, with increases in synaptic proteins such as synapsin-1, postsynaptic density protein 95, and the α-amino-3-hydroxy-5-methyl-4-isoxazolepropionic acid (AMPA) receptor GluA1 subunit appearing as early as two hours after administration, correlating with the onset of ketamine’s antidepressant effects [44,58]. A crucial component of ketamine’s mechanism involves rapid homeostatic synaptic plasticity, specifically through AMPA receptor potentiation, which allows for rapid synaptic scaling, maintaining synaptic stability without disrupting cognitive functions [62]. This type of plasticity adjusts synaptic strengths in response to changes in activity, which may explain ketamine’s immediate and sustained effects on synaptic function and mood regulation [62].

The involvement of adult hippocampal neurogenesis in the antidepressant effects of ketamine is also significant, although the evidence is mixed [44].

Some studies suggest that ketamine accelerates the differentiation of neural progenitor cells into new neurons in the dentate gyrus, which is associated with the drug’s sustained, but not rapid, antidepressant effects [44]. However, other findings suggest that while ketamine may enhance cell proliferation in the dentate gyrus, it does not necessarily promote cell differentiation or maturation into neurons [44]. In the clinical context, a study using diffusion tensor imaging (DTI) monitored microstructural changes in depression-relevant brain regions, such as the amygdala and anterior cingulate cortex, after ketamine administration [63]. The results showed that reduced mean diffusivity (MD), a marker of enhanced neuroplasticity, correlated with improvements in depressive symptoms. This reduction in MD reflects increased synaptogenesis and structural reorganization, which help reverse the neural deficits seen in depression [63].

Moreover, the effects on synaptic plasticity, which also contribute to the long-term antidepressant effect, are partially mediated by epigenetic changes. Key mechanisms include alterations in DNA methylation and histone post-translational modifications (PTMs), particularly in stress-responsive brain regions such as the prefrontal cortex (PFC) and hippocampus. For instance, ketamine reduces the hypermethylation of the BDNF gene promoter, a process often heightened in stress-related conditions, thereby enhancing BDNF transcription and promoting neuroplasticity [64]. Furthermore, ketamine increases histone H3K9 acetylation and facilitates the phosphorylation and cytoplasmic export of histone deacetylase 5 (HDAC5), leading to the upregulation of plasticity-related genes like eIF4EBP1 and CREB, which are essential for synaptic remodeling [64]. These epigenetic modifications play a crucial role in reversing stress-induced neural deficits and sustaining antidepressant effects over time [64].

**Figure 1:**
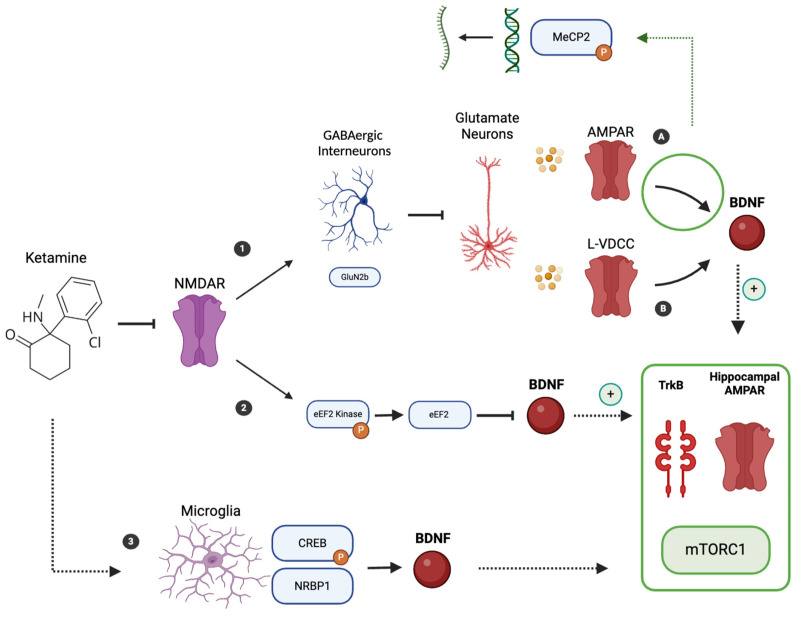
Mechanisms of ketamine action involving brain-derived neurotrophic factor (BDNF). Ketamine acts as an antagonist of the N-methyl-D-aspartate (NMDA) receptor. The antagonism of NMDA receptors (NMDAR) is particularly important at the level of GABAergic interneurons (1), especially those expressing the GluN2b subunit. Indeed, this antagonism leads to a reduction in their activity, which normally inhibits glutamatergic pyramidal neurons (Glutamate Neurons). Consequently, the activity of these pyramidal neurons increases, leading to the release of glutamate, which primarily affects AMPA receptors (AMPAR) (A) and L-type voltage-dependent Ca2+ channels (L-VDCCs) (B), with a subsequent increase in the production of BDNF. Specifically, the activation of AMPAR leads to the phosphorylation of methyl CpG binding protein 2 (MeCP2), resulting in an increased transcription of genes such as BDNF. Additionally, NMDA receptor activation inhibits Eukaryotic elongation factor 2 (eEF2) kinase (2) (eEF2K), which normally phosphorylates eEF2, thereby inhibiting BDNF production. The dephosphorylation of eEF2 thus results in increased BDNF production. Furthermore, microglia (3) also play a role, with ketamine increasing the expression of Nuclear Receptor Binding Protein 1 (NRBP1) and phosphorylated cAMP response element-binding protein (CREB), leading to an increased transcription of BDNF. The increase in BDNF through these mechanisms stimulates the mammalian target of rapamycin complex 1 (mTORC1) signaling pathways, promoting increased protein synthesis and the formation of new dendritic spines. Additionally, this leads to the upregulation of hippocampal AMPAR subunits GluA1 and GluA2, and the activation of tropomyosin receptor kinase B (TrkB), which triggers intracellular pathways that contribute to synaptogenesis and the enhancement of NMDA receptor activity.

Focusing instead on the relationship between ketamine’s antidepressant effects, its effects on synaptogenesis and neural plasticity, and BDNF, we must consider the molecular mechanism of action of ketamine itself (Figure 1), with particular attention to its effect on glutamatergic transmission [44,59]. Ketamine rapidly increases extracellular glutamate levels in the mPFC, a process thought to result from a blockade of NMDA receptors on GABAergic interneurons [44,59]. This leads to disinhibition of these neurons, which enhances activation of postsynaptic AMPA receptors, resulting in depolarization and subsequent activation of L-type voltage-dependent Ca2+ channels (L-VDCCs) [44,65,66,67]. Activation of AMPA receptors and L-VDCCs is essential for the rapid release of BDNF [68].

This has been observed in studies with cultured cells [69], which demonstrated that blocking L-VDCCs prevents the BDNF release stimulated by AMPA receptors [69]. The necessity of AMPA receptors was validated by studies showing that treatment with the antagonist NBQX entirely inhibits the ketamine-induced release of BDNF [69]. Furthermore, pretreatment with the VDCC blocker verapamil completely prevented the BDNF release triggered by ketamine [68].

In addition, ketamine acts through a distinct mechanism on pathways upstream of glutamate release [70]. It specifically targets and inhibits NMDA receptors containing the GluN2b subunit, which are primarily found on GABAergic interneurons [70]. This selective inhibition results in disinhibition of cortical pyramidal neurons, allowing glutamate to flood into the synaptic cleft [70]. Once there, glutamate binds to AMPA receptors as described above [70]. In addition, Eukaryotic elongation factor 2 (eEF2) kinase (eEF2K), also known as CaMKIII, plays a role in the antidepressant effects of ketamine [62]. Ketamine’s effect on NMDA receptors disrupts the basal calcium signals maintained by tonically active NMDA receptors, leading to inhibition of eEF2K and subsequent dephosphorylation of eEF2 [62]. This dephosphorylation de-represses dendritic protein synthesis and specifically promotes the production of BDNF [60,62,71,72].

Unlike traditional monoaminergic antidepressants, which gradually increase BDNF expression, ketamine induces a rapid release of BDNF [73].

**Figure 2:**
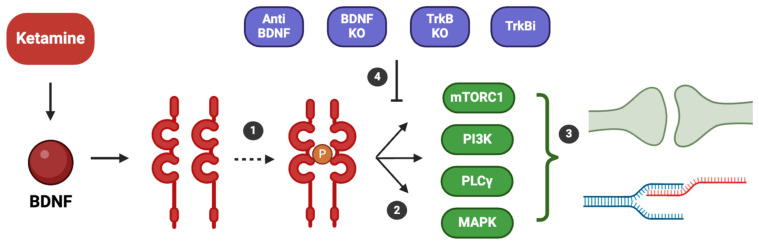
Binding of brain-derived neurotrophic factor (BDNF) to Tropomyosin Receptor Kinase B (TrkB) and the resulting neurotrophic mechanisms involved in depression. When BDNF binds to TrkB, it induces dimerization and autophosphorylation of its tyrosine kinase (1). This phosphorylation activates several intracellular signaling pathways (2), including the mammalian target of rapamycin complex 1 (mTORC1), phosphoinositide 3-kinase (PI3K), phospholipase C gamma (PLCγ), and mitogen-activated protein kinase (MAPK). These pathways regulate processes such as neuroplasticity, increasing cellular transcription and enhancing synaptogenesis (3). The importance of the BDNF-TrkB interaction for ketamine’s antidepressant action is highlighted by the fact that animal models treated with anti-BDNF antibodies, or knockout (KO) models for the BDNF or TrkB gene, or with selective TrkB inhibitors (TrkBi) (4), do not respond to ketamine’s antidepressant effects.

The importance of BDNF and its interaction with TrkB in ketamine’s mechanism of action is highlighted by studies showing that ketamine’s antidepressant effects are blocked by the infusion of an anti-BDNF neutralizing antibody into the mPFC [68], by BDNF or TrkB knockout models, and by the systemic administration of a selective TrkB inhibitor [1,38,62] (Figure 2).

**Figure 3:**
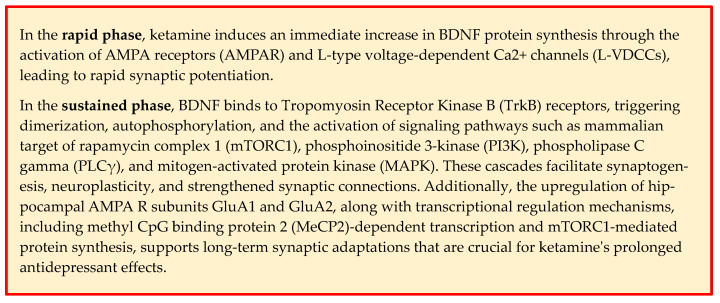
Rapid and sustained mechanisms of ketamine and BDNF on synaptic plasticity.

Additional evidence for the critical role of BDNF in the action of ketamine comes from studies using BDNF Val66Met knock-in mice and conditional BDNF knockout models in which the antidepressant effects of ketamine are significantly reduced [60,74]. These findings are supported by experiments showing that a single infusion of recombinant BDNF into the mPFC produces rapid and sustained antidepressant effects similar to those observed with ketamine administration [75,76]. Thus, ketamine impacts neuroplasticity, including through BDNF, in both a rapid and sustained manner [18,19,77] (Figure 3). The sustained and long-term antidepressant effects of ketamine may be related to initial protein translation-dependent effects, changes in transcriptional regulation initiated by early synaptic plasticity, and the pivotal role of methyl CpG binding protein 2 (MeCP2) [62,78]. MeCP2, a transcriptional regulator involved in synaptic plasticity and neurotransmission, is regulated by activity-dependent phosphorylation at serine 421 (Ser421) [1,51,59]. The AMPA receptor-dependent synaptic potentiation induced by ketamine causes an excitation shift that increases MeCP2 Ser421 phosphorylation, leading to transcriptional changes that support long-term synaptic adaptations essential for sustained antidepressant effects [79]. While MeCP2 Ser421 phosphorylation is required for these long-lasting effects, it is not required for the initial rapid antidepressant response [78,80]. The long-lasting antidepressant effects of (R)-ketamine are increasingly linked to microglial signaling, particularly through the Extracellular signal-regulated kinases (ERK)- Nuclear Receptor Binding Protein 1(NRBP1)- cAMP response element-binding protein (CREB)-BDNF pathway [81]. Microglia, which were traditionally considered passive support cells, are now recognized as active players in synaptic plasticity and mood regulation [82]. Upon administration of (R)-ketamine, there is a marked increase in the expression of NRBP1 and the phosphorylation of CREB within microglial cells [81]. This phosphorylation is a key step in upregulating BDNF transcription, a pivotal factor in mediating synaptic plasticity and promoting resilience against stress-related depressive behaviors [81]. The involvement of microglia extends beyond mere signaling intermediaries [82]. Studies using pharmacological inhibitors or genetic ablation models of microglia have demonstrated that the absence or inhibition of these cells completely abolishes the antidepressant effects of ketamine, emphasizing their essential role in the drug’s mechanism of action [81]. For example, microglial activation through the ERK pathway not only facilitates BDNF production but also modulates inflammatory cytokines and neuroimmune signaling, processes that are often dysregulated in depression [65,81]. Additionally, (R)-ketamine’s effect on microglia has been shown to involve suppression of pro-inflammatory mediators, such as interleukin-6 (IL-6) and tumor necrosis factor-alpha (TNF-α), which contribute to neuroinflammation [81,83]. This anti-inflammatory property of (R)-ketamine may enhance the functional role of microglia in supporting neuronal health, reducing oxidative stress, and promoting synaptic connectivity [81,83]. Furthermore, (R)-ketamine suppresses the transcriptional repressor MeCP2, further promoting BDNF expression, which is vital for its sustained antidepressant effects [81]. Regarding the connection between neuronal and glial BDNF, some studies have shown that neuronal BDNF prevents microglia from engulfing mossy fiber synapses in the hippocampus [84] and that BDNF is generally essential for the normal development of glial cells [85].

The rapid phase of ketamine’s antidepressant action involves an immediate increase in BDNF protein synthesis, which drives downstream intracellular signaling through MeCP2. BDNF then activates TrkB receptors on postsynaptic neurons and increases surface expression of the hippocampal AMPA receptor subunits GluA1 and GluA2, which are necessary for the synaptic and behavioral effects of ketamine [49,52,59,60]. Upon binding to TrkB receptors, BDNF triggers receptor dimerization and autophosphorylation of its tyrosine kinase domain. This phosphorylation event activates multiple intracellular signaling cascades, including the mammalian target of rapamycin complex 1 (mTORC1), phosphoinositide 3-kinase (PI3K), phospholipase C gamma (PLCγ), and mitogen-activated protein kinase (MAPK) pathways [86,87]. These pathways play a crucial role in regulating neuroplasticity by promoting cellular transcription, facilitating synaptogenesis, and strengthening synaptic connections. The importance of BDNF and its interaction with TrkB in ketamine’s mechanism of action is highlighted by studies showing that ketamine’s antidepressant effects are blocked by the infusion of an anti-BDNF neutralizing antibody into the mPFC [68], by BDNF or TrkB knockout models, and by the systemic administration of a selective TrkB inhibitor [1,38,62] (Figure 2).

More specifically, one result of BDNF-TrkB activation by ketamine is stimulation of mTORC signaling pathway [86,87]: this is critical for synaptic protein synthesis and new dendritic spine formation, processes that are fundamental to the sustained antidepressant effects of ketamine [58,60]. mTORC1 regulates the translation of synaptic proteins, such as synapsin-1 and PSD-95, which are essential for maintaining synaptic stability and remodeling [88]. Research suggests that ketamine’s activation of mTORC1 occurs downstream of BDNF signaling and is not directly linked to its initial synaptic engagement [89]. While mTOR activation is crucial for ketamine’s sustained antidepressant effects, it likely does not contribute to the rapid antidepressant response [89]. Instead, mTORC1 activation appears to be driven by BDNF signaling, and in turn, mTORC1 itself might enhance BDNF production, creating a positive feedback loop that sustains neuroplasticity [75,76]. This is supported by clinical findings indicating that rapamycin, an mTOR inhibitor, does not reduce the rapid antidepressant effects of ketamine and may actually enhance them [62]. Furthermore, mTORC1 plays a critical role in local synaptic protein synthesis and cytoskeletal reorganization, such as actin polymerization, enabling the formation and stabilization of new synaptic connections [90,91]. mTORC1 signaling may also be linked to intracellular pathways that activate downstream transcriptional processes involving MeCP2, which are essential for long-term synaptic adaptations and sustained antidepressant effects [62]. Additionally, BDNF may contribute to ketamine’s antidepressant effects through other molecules, such as the neuropeptide VGF (non-acronym) [92]. VGF, which is not an acronym, is upregulated by exercise and ketamine but downregulated by stress, and it increases in the prefrontal cortex after ketamine administration [92]. Blocking VGF reduces ketamine-induced mTOR signaling and diminishes its antidepressant effects, while overexpression of VGF prevents stress-induced behavioral deficits [92]. VGF knockout mice are more vulnerable to stress after ketamine exposure, indicating that VGF plays a key role in ketamine’s rapid effects through BDNF [92].

The neurotrophic potential of ketamine has even led to studies exploring its application in patients with neurodegenerative diseases such as Alzheimer’s [93]. In fact, recent clinical findings suggest that ketamine may provide neuroprotection and alleviate neuropsychiatric symptoms associated with Alzheimer’s disease, due to its ability to act as an NMDA receptor antagonist and modulate brain inflammation and glutamate-related neurotoxicity [93].

Ketamine acts as an antagonist of the N-methyl-D-aspartate (NMDA) receptor. The antagonism of NMDA receptors (NMDAR) is particularly important at the level of GABAergic interneurons (1), especially those expressing the GluN2b subunit. Indeed, this antagonism leads to a reduction in their activity, which normally inhibits glutamatergic pyramidal neurons (Glutamate Neurons). Consequently, the activity of these pyramidal neurons increases, leading to the release of glutamate, which primarily affects AMPA receptors (AMPAR) (A) and L-type voltage-dependent Ca2+ channels (L-VDCCs) (B), with a subsequent increase in the production of BDNF. Specifically, the activation of AMPAR leads to the phosphorylation of methyl CpG binding protein 2 (MeCP2), resulting in an increased transcription of genes such as BDNF. Additionally, NMDA receptor activation inhibits Eukaryotic elongation factor 2 (eEF2) kinase (eEF2K), which normally phosphorylates eEF2, thereby inhibiting BDNF production. The dephosphorylation of eEF2 thus results in increased BDNF production. Furthermore, microglia (3) also play a role, with ketamine increasing the expression of Nuclear Receptor Binding Protein 1 (NRBP1) and phosphorylated cAMP response element-binding protein (CREB), leading to an increased transcription of BDNF. The increase in BDNF through these mechanisms stimulates the mammalian target of rapamycin complex 1 (mTORC1) signaling pathways, promoting increased protein synthesis and the formation of new dendritic spines. Additionally, this leads to the upregulation of hippocampal AMPAR subunits GluA1 and GluA2, and the activation of tropomyosin receptor kinase B (TrkB), which triggers intracellular pathways that contribute to synaptogenesis and the enhancement of NMDA receptor activity.

## 5. Evidence for the Effects of Ketamine and Esketamine on BDNF Levels in Humans

### 5.1. Ketamine

Several studies have focused on the effects of ketamine on BDNF levels, with mixed results (Table 1). For example, one study examined plasma BDNF levels in 22 patients with TRD who were randomized to receive either intravenous ketamine at 0.5 mg/kg or intravenous midazolam at 0.045 mg/kg. The study found that ketamine significantly increased plasma BDNF levels in responders compared to non-responders, with measurements taken 240 min after infusion [94]. Similarly, a study showed that a single intravenous infusion of 0.5 mg/kg ketamine was associated with significantly increased plasma BDNF levels at 230 min post-infusion compared with baseline measurements [95].

A separate study [96] examined the relationship between ketamine-induced changes in plasma BDNF levels and resting-state functional connectivity (RSFC) of the prefrontal cortex in 53 healthy participants. In this randomized, placebo-controlled study, ketamine was administered at a subanesthetic dose of 0.5 mg/kg via a continuous 40 min infusion. Participants received a single infusion, with effects on plasma BDNF levels and RSFC measured acutely at 120 min post-infusion and again at 24 h. The study found that ketamine administration resulted in significant increases in plasma BDNF levels at both 2 h and 24 h post-infusion. These BDNF changes were associated with decreased RSFC between the dorsomedial prefrontal cortex (dmPFC) and regions such as the posterior cingulate cortex (PCC) and ventromedial prefrontal cortex (vmPFC). The RSFC changes were more pronounced in participants who showed an increase in BDNF levels after ketamine, suggesting that the antidepressant effects of ketamine may be related to enhanced synaptic plasticity, reflected in both BDNF levels and RSFC changes [96].

However, some studies have not observed these effects. For instance, one study of 23 TRD patients who received open-label intravenous ketamine (0.5 mg/kg) reported no increase in plasma BDNF levels [97]. In addition, another study of patients with MDD without psychotic features who had not responded to at least one adequate antidepressant trial and who received intravenous ketamine at 0.5 mg/kg found no increase in plasma BDNF levels 230 min after infusion [98].

Adding to this complexity, a study comparing serum BDNF levels in TRD patients treated with intravenous ketamine (0.5 mg/kg) or esketamine (0.25 mg/kg) found no significant differences at baseline, 24 h, or 72 h post-treatment [20].

Other studies have instead considered BDNF as a potential predictor of specific symptoms or traits. For example, one study reported that repeated intravenous administration of ketamine had significant anti-anhedonic effects in patients with MDD, particularly in those with higher baseline plasma BDNF levels. This study involved 75 Chinese patients and found that individuals with higher plasma BDNF levels at baseline experienced a greater reduction in anhedonia following ketamine treatment than those with lower baseline plasma BDNF levels [100]. Another study of 127 patients with MDD disorder or bipolar disorder examined the effects of repeated ketamine infusions on sleep quality and its correlation with antidepressant outcomes. The study showed significant improvements in both sleep disturbance and depression scores after ketamine infusions. Patients who responded to sleep improvements had significantly higher BDNF levels than non-responders [101]. A study comparing ketamine to midazolam in patients with bipolar depression and significant suicidal ideation showed that serum BDNF levels decreased from pre- to post-infusion in both treatment groups. However, a significant correlation was found between the reduction in suicidal ideation and the decrease in BDNF levels after ketamine infusion, but not after midazolam infusion [102].

It is challenging to explain the differences in the results obtained from the studies mentioned above. First, the small sample sizes across all these studies make it difficult to generalize the findings. This limitation in statistical power might explain some of the inconsistencies in the outcomes. Second, several authors have highlighted that the optimal time for measuring BDNF levels remains unclear. Depending on the timing of the measurements, studies have reported different results. As noted, the studies cited above do not always use the same time intervals for BDNF measurements, which may contribute to the variability in findings [20]. This discrepancy makes it hard to establish a clear consensus on how ketamine influences BDNF. Moreover, some of the results presented are derived from secondary analyses of previously conducted studies [80,83]; therefore, the setting may not be optimal for the objective considered (examining BDNF variations in patients treated with ketamine). In any case, it might be useful to consider other potential biomarkers, such as 5-HT [103], as highlighted in the study by Wang et al., where ketamine and esketamine were indeed found to have an impact on its increase [99].

### 5.2. Esketamine

Research on esketamine also shows its potential effect on BDNF levels. The results we found in the literature regarding BDNF changes in humans treated with esketamine are mostly from studies of specific populations (Table 2): one study reported that intravenous esketamine at a dose of 0.25 mg/kg significantly increased plasma BDNF levels in patients with postpartum depression, with this increase measured three days after infusion [104]. Another study found that intravenous administration of esketamine (0.5 mg/kg) effectively reduced anxiety and depression in patients undergoing thoracic surgery. This reduction in symptoms was associated with increased serum levels of BDNF [105]. In addition, a study of 417 patients with cervical cancer undergoing laparoscopic modified radical hysterectomy found that those who received 0.5 mg/kg intravenous esketamine during surgery had greater increases in BDNF levels in the days following surgery compared to patients treated with placebo, ketamine, or esketamine at a dose of 0.25 mg/kg [99].

## 6. Discussion

The role of BDNF in the pathophysiology of MDD and the therapeutic mechanisms of antidepressants has been extensively studied [2,22]. BDNF is critical for synaptic plasticity, neurogenesis, and neuronal survival, and its altered expression has been implicated in the development of depressive symptom [2,22]. The neurotrophic hypothesis of depression suggests that reduced BDNF levels in key brain regions, such as the hippocampus and prefrontal cortex, contribute to the neuronal atrophy and synaptic dysfunction observed in MDD, which in turn leads to the manifestation of depressive symptoms [2,3]. Conventional antidepressants, particularly SSRIs and SNRIs, are known to increase BDNF levels over time, which correlates with their delayed therapeutic effect [3,41]. However, the need for treatments that provide more rapid relief has focused attention on ketamine and esketamine, both of which have demonstrated the ability to induce fast-acting antidepressant effects [13,16,17].

The mechanism of action of ketamine primarily involves glutamatergic pathways at the molecular level, mediated by NMDA receptor antagonism and AMPA receptor activation. While these glutamatergic changes explain the rapid action of ketamine, its medium- to long-term effects must be attributed to other mechanisms [20]. In particular, ketamine has been shown in preclinical studies to induce neuroplasticity and synaptogenesis [18,19]. Experimental evidence suggests a strong involvement of BDNF in these processes [18,19]. However, studies in humans are still limited, in part due to methodological challenges and the relatively recent approval of esketamine by several international agencies for the treatment of TRD. In the literature, an increase in plasma BDNF has been reported in depressed patients treated with ketamine. For esketamine, studies have been conducted in special populations, such as patients with postpartum depression [104] or those who have undergone surgery [99,105]. The small sample sizes of these studies, combined with the heterogeneity of the populations studied, may partly explain the variability in the results observed. In addition, it is important to note that the central neurotrophic mechanisms involving BDNF are studied experimentally with ketamine, whereas human studies typically focus on plasma BDNF levels. Furthermore, an important gap in the literature is the effect of long-term ketamine and esketamine treatments on BDNF levels, as, to our knowledge, no clinical studies have addressed this aspect.

Considering the potential connection between ketamine, esketamine, their antidepressant effects, and BDNF, it is essential to also examine GLYX-13 (Rapastinel). Like ketamine and esketamine, GLYX-13 exerts its antidepressant effects by targeting the glutamatergic system, specifically through NMDA receptor modulation [76]. Its antidepressant actions are mediated by the activity-dependent release of BDNF, which plays a crucial role in enhancing synaptic plasticity [76]. Blocking BDNF-TrkB signaling, whether through neutralizing antibodies or genetic models that inhibit BDNF release, has been shown to completely eliminate the antidepressant effects of GLYX-13 [76].

In discussing the potential link between BDNF, ketamine/esketamine, and their antidepressant effects, given that the studies mentioned above focus on plasma BDNF, it is also important to consider the use of plasma BDNF as a proxy for central BDNF activity. Several studies have investigated the relationship between peripheral BDNF levels and central BDNF activity, contributing to the ongoing debate regarding the use of BDNF as a biomarker for brain function. It has been demonstrated that in rats and pigs, BDNF levels in blood and plasma correlate with hippocampal BDNF levels, supporting the potential use of peripheral BDNF as a biomarker for brain-derived BDNF [106]. However, this correlation is less clear in the frontal cortex, and significant species differences in BDNF transport and detection have been observed, particularly in mice, where blood BDNF is undetectable despite measurable brain BDNF levels [106]. Moreover, a correlation between serum BDNF and cortical BDNF levels was found in young rats, although this relationship weakens with age [107]. These findings were further supported by a study demonstrating a significant correlation between serum and brain BDNF levels following electroconvulsive therapy, particularly in regions involved in mood regulation, such as the hippocampus and prefrontal cortex [108]. Nonetheless, discrepancies exist, as an inverse relationship between blood and hippocampal BDNF levels was observed in a genetic rat model of depression, raising questions about the universality of these correlations [109]. The capacity of BDNF to cross the blood-brain barrier adds further complexity, as it has been shown that BDNF can indeed cross the barrier in mice [110].

In addition, as previously mentioned, there are regional variations in BDNF expression [2]. For example, in the hippocampus, particularly in the dentate gyrus and CA3 regions, increased BDNF promotes neurogenesis and synaptic plasticity, leading to enhanced mood regulation [2]. In the mPFC, BDNF strengthens synaptic connectivity, supporting both rapid and long-term synaptic adaptations [2]. Conversely, elevated BDNF in the nucleus accumbens has been linked to increased stress vulnerability and depression-like behaviors, while reduced BDNF in this region is associated with resilience to stress [35]. In the ventral tegmental area [111], BDNF influences reward-related behavior, playing a role in both resilience and susceptibility to depression. However, there is limited evidence on regional variations in BDNF induced by antidepressants, with most findings highlighting effects in the hippocampus and PFC [44]. It is also crucial to mention the well-established action of ketamine on BDNF levels in the prefrontal cortex, which plays a significant role in its antidepressant effects [44]. Future studies are needed to clarify the impact of ketamine and esketamine on BDNF levels in various brain regions, as it has been demonstrated that these levels differ from one area to another. In any case, measuring plasma BDNF is currently the most practical approach. Nevertheless, studies such as the one mentioned above, which relate ketamine treatment and its antidepressant effect not only to changes in BDNF levels but also to RSFC, are of great interest [96].

## 7. Conclusions

In conclusion, BDNF has emerged as a key factor in the pathophysiology of depression and the therapeutic effects of antidepressants, particularly ketamine and esketamine. Its role in promoting neuroplasticity, synaptogenesis, and neurogenesis underscores its importance in the rapid and sustained antidepressant effects observed with these treatments. While evidence from preclinical studies supports the link between BDNF, depression, and the mechanisms of action of antidepressants, data from human studies remain inconclusive, limited to a few investigations with varying designs and often insufficiently large samples. Further studies are needed, particularly for esketamine and especially for the formulation of esketamine currently approved for TRD, i.e., the nasal spray [12], to better understand the relationship between BDNF and the antidepressant effect of ketamine. While the connection between BDNF and traditional antidepressants is well established, clinical studies are particularly necessary to explore the link between BDNF levels in human patients and the antidepressant effects of ketamine or esketamine. Additionally, further preclinical studies are required to investigate whether there are differences in the neuroplasticity pathways modulated by esketamine and ketamine (even though it is worth noting that esketamine is the S-enantiomer of ketamine and, thus, ketamine is a racemic mixture containing both esketamine and arketamine). It will also be of interest to use other tools to understand potential neuroplasticity effects, such as RSFC studies. The study of BDNF is useful not only in the search for potential biomarkers for the improvement of psychiatric disorders, but also as a direct indicator of the importance of neuroplasticity and neurotrophism for the understanding and treatment of depression.

## Figures and Tables

**Table 1:** Clinical studies on the effects of ketamine on BDNF variations in human patients. TRD: treatment resistant depression; MDD: major depressive disorder.

Authors	Study Design	Time of Measurement	Outcome—Ketamine
Haile, et al. [94]	44 patients with TRD treated with IV Ketamine (0.5 mg/kg) or IV Midazolam (0.045 mg/kg)	240 min post-infusion	Ketamine significantly increased plasma BDNF in responders compared to non-responders
Duncan Jr, et al. [95]	30 patients with TRD treated with IV Ketamine (0.5 mg/kg)	230 min post-infusion	Ketamine significantly increased plasma BDNF
Woelfer, et al. [96]	80 healthy volunteers treated with IV Ketamine (0.5 mg/kg) or IV NaCl 0.9%	120 min and 24 h post-infusion	Ketamine significantly increased plasma BDNF at both time points compared to placebo
Machado-Vieira, et al. [97]	23 patients with TRD treated with IV Ketamine (0.5 mg/kg)	40, 80, 120, and 230 min post-infusion	No significant increase in plasma BDNF at any time point
Medeiros, et al. [98]	39 patients with major depressive disorder (MDD) treated with IV Ketamine (0.5 mg/kg) or IV saline	230 min, 24 h, and 72 h post-infusion	No significant increase in plasma BDNF at any time point compared to placebo
Wang, et al. [99]	417 cervical carcinoma patients receiving 0.25 mg/kg IV esketamine or 0.5 mg/kg IV esketamine or 0.5 mg/kg IV ketamine or IV saline	24, 48, 72, 120 and 168 h after the surgery	Ketamine significantly increased plasma BDNF at 24, 48 and 72 h compared to placebo
Caliman-Fontes, et al. [20]	53 patients with TRD treated with IV Ketamine (0.5 mg/kg) or IV Esketamine (0.25 mg/kg)	24 h and 168 h (1 week) post-infusion	No significant increase in plasma BDNF at any time point

**Table 2:** Clinical studies on the effects of esketamine on BDNF variations in human patients. TRD: Treatment resistant depression.

Authors	Study Design	Time of Measurement	Outcome—Esketamine
Jiang, et al. [104]	315 patients with postpartum depression treated with IV esketamine (0.25 mg/kg) or IV saline	72 h post-infusion	Esketamine significantly increased plasma BDNF compared to placebo
Luo, et al. [105]	129 adult patients that underwent elective non-cardiac thoracic surgery under general anesthesia treated with 0.2 mg/kg IV esketamine or 0.5 mg/kg IV esketamine or with IV saline	End of the surgery and three days after the surgery	0.5 mg/kg esketamine significantly increased plasma BDNF compared to placebo
Wang, et al. [99]	417 cervical carcinoma patients receiving 0.25 mg/kg IV esketamine or 0.5 mg/kg IV esketamine or 0.5 mg/kg IV ketamine or IV saline	24, 48, 72, 120 and 168 h after the surgery	Both 0.25 mg/kg esketamine and 0.5 mg/kg esketamine significantly increased plasma BDNF at 24, 48 and 72 h compared to placebo
Caliman-Fontes, et al. [20]	53 patients with TRD treated with IV Ketamine (0.5 mg/kg) or IV Esketamine (0.25 mg/kg)	24 h and 168 h (1 week) post-infusion	No significant increase in plasma BDNF at any time point

## References

[1] Hasler G. (2010). Pathophysiology of Depression: Do We Have Any Solid Evidence of Interest to Clinicians?. World Psychiatry.

[2] Duman R.S., Deyama S., Fogaça M.V. (2021). Role of BDNF in the Pathophysiology and Treatment of Depression: Activity-Dependent Effects Distinguish Rapid-Acting Antidepressants. Eur. J. Neurosci..

[3] Cavaleri D., Moretti F., Bartoccetti A., Mauro S., Crocamo C., Carrà G., Bartoli F. (2023). The Role of BDNF in Major Depressive Disorder, Related Clinical Features, and Antidepressant Treatment: Insight from Meta-Analyses. Neurosci. Biobehav. Rev..

[4] Miyanishi H., Nitta A. (2021). A Role of BDNF in the Depression Pathogenesis and a Potential Target as Antidepressant: The Modulator of Stress Sensitivity “Shati/Nat8l-BDNF System” in the Dorsal Striatum. Pharmaceuticals.

[5] Yu H., Chen Z. (2011). The Role of BDNF in Depression on the Basis of Its Location in the Neural Circuitry. Acta Pharmacol. Sin..

[6] Yang T., Nie Z., Shu H., Kuang Y., Chen X., Cheng J., Yu S., Liu H. (2020). The Role of BDNF on Neural Plasticity in Depression. Front. Cell. Neurosci..

[7] Mosiołek A., Mosiołek J., Jakima S., Pięta A., Szulc A. (2021). Effects of Antidepressant Treatment on Neurotrophic Factors (BDNF and IGF-1) in Patients with Major Depressive Disorder (MDD). J. Clin. Med..

[8] McIntyre R.S., Alsuwaidan M., Baune B.T., Berk M., Demyttenaere K., Goldberg J.F., Gorwood P., Ho R., Kasper S., Kennedy S.H. (2023). Treatment-Resistant Depression: Definition, Prevalence, Detection, Management, and Investigational Interventions. World Psychiatry.

[9] Gaynes B.N., Lux L., Gartlehner G., Asher G., Forman-Hoffman V., Green J., Boland E., Weber R.P., Randolph C., Bann C. (2020). Defining Treatment-Resistant Depression. Depress. Anxiety.

[10] Demyttenaere K., Van Duppen Z. (2019). The Impact of (the Concept of) Treatment-Resistant Depression: An Opinion Review. Int. J. Neuropsychopharmacol..

[11] Pigott H.E., Kim T., Xu C., Kirsch I., Amsterdam J. (2023). What Are the Treatment Remission, Response and Extent of Improvement Rates After up to Four Trials of Antidepressant Therapies in Real-World Depressed Patients? A Reanalysis of the STAR*D Study’s Patient-Level Data with Fidelity to the Original Research Protocol. BMJ Open.

[12] Food and Drug Administration (FDA) (2019). FDA Approves New Nasal Spray Medication for Treatment-Resistant Depression, Available Only at a Certified Doctor’s Office or Clinic.

[13] Vasiliu O. (2023). Esketamine for Treatment-Resistant Depression: A Review of Clinical Evidence (Review). Exp. Ther. Med..

[14] Swainson J., Thomas R.K., Archer S., Chrenek C., MacKay M.-A., Baker G., Dursun S., Klassen L.J., Chokka P., Demas M.L. (2019). Esketamine for Treatment Resistant Depression. Expert Rev. Neurother..

[15] Dean R.L., Hurducas C., Hawton K., Spyridi S., Cowen P.J., Hollingsworth S., Marquardt T., Barnes A., Smith R., McShane R. (2021). Ketamine and Other Glutamate Receptor Modulators for Depression in Adults with Unipolar Major Depressive Disorder. Cochrane Database Syst. Rev..

[16] Castro M., Wilkinson S.T., Al Jurdi R.K., Petrillo M.P., Zaki N., Borentain S., Fu D.J., Turkoz I., Sun L., Brown B. (2023). Efficacy and Safety of Esketamine Nasal Spray in Patients with Treatment-Resistant Depression Who Completed a Second Induction Period: Analysis of the Ongoing SUSTAIN-3 Study. CNS Drugs.

[17] McIntyre R.S., Carvalho I.P., Lui L.M.W., Majeed A., Masand P.S., Gill H., Rodrigues N.B., Lipsitz O., Coles A.C., Lee Y. (2020). The Effect of Intravenous, Intranasal, and Oral Ketamine in Mood Disorders: A Meta-Analysis. J. Affect. Disord..

[18] Yavi M., Lee H., Henter I.D., Park L.T., Zarate C.A. (2022). Ketamine Treatment for Depression: A Review. Discov. Ment. Health.

[19] Björkholm C., Monteggia L.M. (2016). BDNF—A Key Transducer of Antidepressant Effects. Neuropharmacology.

[20] Caliman-Fontes A.T., Leal G., Correia-Melo F.S., Paixão C.S., Carvalho M.S., Jesus-Nunes A.P., Vieira F., Magnavita G., Bandeira I.D., Mello R.P. (2023). Brain-Derived Neurotrophic Factor Serum Levels Following Ketamine and Esketamine Intervention for Treatment-Resistant Depression: Secondary Analysis from a Randomized Trial. Trends Psychiatry Psychother..

[21] Binder D.K., Scharfman H.E. (2004). Brain-Derived Neurotrophic Factor. Growth Factors.

[22] Autry A.E., Monteggia L.M. (2012). Brain-Derived Neurotrophic Factor and Neuropsychiatric Disorders. Pharmacol. Rev..

[23] Roux P. (2002). Neurotrophin Signaling Through the P75 Neurotrophin Receptor. Prog. Neurobiol..

[24] Eggert S., Kins S., Endres K., Brigadski T. (2022). Brothers in Arms: ProBDNF/BDNF and sAPPα/Aβ-Signaling and Their Common Interplay with ADAM10, TrkB, p75NTR, Sortilin, and sorLA in the Progression of Alzheimer’s Disease. Biol. Chem..

[25] Levine E.S., Crozier R.A., Black I.B., Plummer M.R. (1998). Brain-Derived Neurotrophic Factor Modulates Hippocampal Synaptic Transmission by Increasing *N*-Methyl-d-Aspartic Acid Receptor Activity. Proc. Natl. Acad. Sci. USA.

[26] Yoshii A., Constantine-Paton M. (2007). BDNF Induces Transport of PSD-95 to Dendrites Through PI3K-AKT Signaling After NMDA Receptor Activation. Nat. Neurosci..

[27] Miranda M., Morici J.F., Zanoni M.B., Bekinschtein P. (2019). Brain-Derived Neurotrophic Factor: A Key Molecule for Memory in the Healthy and the Pathological Brain. Front. Cell. Neurosci..

[28] Bathina S., Das U.N. (2015). Brain-Derived Neurotrophic Factor and Its Clinical Implications. Arch. Med. Sci..

[29] Karpova N.N. (2014). Role of BDNF Epigenetics in Activity-Dependent Neuronal Plasticity. Neuropharmacology.

[30] Hashimoto K., Shimizu E., Iyo M. (2004). Critical Role of Brain-Derived Neurotrophic Factor in Mood Disorders. Brain Res. Rev..

[31] Gao L., Zhang Y., Sterling K., Song W. (2022). Brain-Derived Neurotrophic Factor in Alzheimer’s Disease and Its Pharmaceutical Potential. Transl. Neurodegener..

[32] Nieto R.R., Carrasco A., Corral S., Castillo R., Gaspar P.A., Bustamante M.L., Silva H. (2021). BDNF as a Biomarker of Cognition in Schizophrenia/Psychosis: An Updated Review. Front. Psychiatry.

[33] Bremner J.D., Narayan M., Anderson E.R., Staib L.H., Miller H.L., Charney D.S. (2000). Hippocampal Volume Reduction in Major Depression. Am. J. Psychiatry.

[34] Krishnan V., Han M.-H., Graham D.L., Berton O., Renthal W., Russo S.J., LaPlant Q., Graham A., Lutter M., Lagace D.C. (2007). Molecular Adaptations Underlying Susceptibility and Resistance to Social Defeat in Brain Reward Regions. Cell.

[35] Tebartz Van Elst L., Woermann F., Lemieux L., Trimble M.R. (2000). Increased Amygdala Volumes in Female and Depressed Humans. A Quantitative Magnetic Resonance Imaging Study. Neurosci. Lett..

[36] Frodl T., Meisenzahl E., Zetzsche T., Bottlender R., Born C., Groll C., Jäger M., Leinsinger G., Hahn K., Möller H.-J. (2002). Enlargement of the Amygdala in Patients with a First Episode of Major Depression. Biol. Psychiatry.

[37] Castrén E., Kojima M. (2017). Brain-Derived Neurotrophic Factor in Mood Disorders and Antidepressant Treatments. Neurobiol. Dis..

[38] Molendijk M.L., Spinhoven P., Polak M., Bus B.A.A., Penninx B.W.J.H., Elzinga B.M. (2014). Serum BDNF Concentrations as Peripheral Manifestations of Depression: Evidence from a Systematic Review and Meta-Analyses on 179 Associations (N=9484). Mol. Psychiatry.

[39] Liu X., Li P., Ma X., Zhang J., Sun X., Luo X., Zhang Y. (2022). Association Between Plasma Levels of BDNF and GDNF and the Diagnosis, Treatment Response in First-Episode MDD. J. Affect. Disord..

[40] Zelada M.I., Garrido V., Liberona A., Jones N., Zúñiga K., Silva H., Nieto R.R. (2023). Brain-Derived Neurotrophic Factor (BDNF) as a Predictor of Treatment Response in Major Depressive Disorder (MDD): A Systematic Review. Int. J. Mol. Sci..

[41] Cubillos S., Engmann O., Brancato A. (2022). BDNF as a Mediator of Antidepressant Response: Recent Advances and Lifestyle Interactions. Int. J. Mol. Sci..

[42] Nibuya M., Nestler E., Duman R. (1996). Chronic Antidepressant Administration Increases the Expression of cAMP Response Element Binding Protein (CREB) in Rat Hippocampus. J. Neurosci..

[43] Nibuya M., Morinobu S., Duman R. (1995). Regulation of BDNF and trkB mRNA in Rat Brain by Chronic Electroconvulsive Seizure and Antidepressant Drug Treatments. J. Neurosci..

[44] Deyama S., Duman R.S. (2020). Neurotrophic Mechanisms Underlying the Rapid and Sustained Antidepressant Actions of Ketamine. Pharmacol. Biochem. Behav..

[45] Shirayama Y., Chen A.C.-H., Nakagawa S., Russell D.S., Duman R.S. (2002). Brain-Derived Neurotrophic Factor Produces Antidepressant Effects in Behavioral Models of Depression. J. Neurosci..

[46] Castrén E., Monteggia L.M. (2021). Brain-Derived Neurotrophic Factor Signaling in Depression and Antidepressant Action. Biol. Psychiatry.

[47] Duclot F., Kabbaj M. (2015). Epigenetic Mechanisms Underlying the Role of Brain-Derived Neurotrophic Factor in Depression and Response to Antidepressants. J. Exp. Biol..

[48] Boulle F., Van Den Hove D.L.A., Jakob S.B., Rutten B.P., Hamon M., Van Os J., Lesch K.-P., Lanfumey L., Steinbusch H.W., Kenis G. (2012). Epigenetic Regulation of the BDNF Gene: Implications for Psychiatric Disorders. Mol. Psychiatry.

[49] Chen K.-W., Chen L. (2017). Epigenetic Regulation of BDNF Gene During Development and Diseases. Int. J. Mol. Sci..

[50] Hing B., Davidson S., Lear M., Breen G., Quinn J., McGuffin P., MacKenzie A. (2012). A Polymorphism Associated with Depressive Disorders Differentially Regulates Brain Derived Neurotrophic Factor Promoter IV Activity. Biol. Psychiatry.

[51] Peña C.J., Nestler E.J. (2018). Progress in Epigenetics of Depression. Progress in Molecular Biology and Translational Science.

[52] Chen Z.-Y., Bath K., McEwen B., Hempstead B., Lee F. (2008). Impact of Genetic Variant BDNF (Val66Met) on Brain Structure and Function. Novartis Found Symp..

[53] Colle R., Gressier F., Verstuyft C., Deflesselle E., Lépine J.-P., Ferreri F., Hardy P., Guilloux J.-P., Petit A.-C., Fève B. (2015). Brain-Derived Neurotrophic Factor Val66Met Polymorphism and 6-Month Antidepressant Remission in Depressed Caucasian Patients. J. Affect. Disord..

[54] Smit A.J.T., Wu G.W.Y., Rampersaud R., Reus V.I., Wolkowitz O.M., Mellon S.H. (2024). Serum Brain-Derived Neurotrophic Factor, Val66Met Polymorphism and Open-Label SSRI Treatment Response in Major Depressive Disorder. Psychoneuroendocrinology.

[55] Yan T., Wang L., Kuang W., Xu J., Li S., Chen J., Yang Y. (2014). Brain-Derived Neurotrophic Factor Val66Met Polymorphism Association with Antidepressant Efficacy: A Systematic Review and Meta-Analysis: BDNF Val66Met and Antidepressant Efficacy. Asia-Pac. Psychiatry.

[56] Hirota K., Lambert D.G. (2022). Ketamine; History and Role in Anesthetic Pharmacology. Neuropharmacology.

[57] Mion G., Villevieille T. (2013). Ketamine Pharmacology: An Update (Pharmacodynamics and Molecular Aspects, Recent Findings). CNS Neurosci. Ther..

[58] Li N., Lee B., Liu R.-J., Banasr M., Dwyer J.M., Iwata M., Li X.-Y., Aghajanian G., Duman R.S. (2010). mTOR-Dependent Synapse Formation Underlies the Rapid Antidepressant Effects of NMDA Antagonists. Science.

[59] Zanos P., Gould T.D. (2018). Mechanisms of Ketamine Action as an Antidepressant. Mol. Psychiatry.

[60] Autry A.E., Adachi M., Nosyreva E., Na E.S., Los M.F., Cheng P., Kavalali E.T., Monteggia L.M. (2011). NMDA Receptor Blockade at Rest Triggers Rapid Behavioural Antidepressant Responses. Nature.

[61] Moda-Sava R.N., Murdock M.H., Parekh P.K., Fetcho R.N., Huang B.S., Huynh T.N., Witztum J., Shaver D.C., Rosenthal D.L., Alway E.J. (2019). Sustained Rescue of Prefrontal Circuit Dysfunction by Antidepressant-Induced Spine Formation. Science.

[62] Krystal J.H., Kavalali E.T., Monteggia L.M. (2024). Ketamine and Rapid Antidepressant Action: New Treatments and Novel Synaptic Signaling Mechanisms. Neuropsychopharmacology.

[63] Kopelman J., Keller T.A., Panny B., Griffo A., Degutis M., Spotts C., Cruz N., Bell E., Do-Nguyen K., Wallace M.L. (2023). Rapid Neuroplasticity Changes and Response to Intravenous Ketamine: A Randomized Controlled Trial in Treatment-Resistant Depression. Transl. Psychiatry.

[64] Inserra A., Campanale A., Rezai T., Romualdi P., Rubino T. (2024). Epigenetic Mechanisms of Rapid-Acting Antidepressants. Transl. Psychiatry.

[65] Duman R.S., Aghajanian G.K., Sanacora G., Krystal J.H. (2016). Synaptic Plasticity and Depression: New Insights from Stress and Rapid-Acting Antidepressants. Nat. Med..

[66] Maeng S., Zarate C.A., Du J., Schloesser R.J., McCammon J., Chen G., Manji H.K. (2008). Cellular Mechanisms Underlying the Antidepressant Effects of Ketamine: Role of α-Amino-3-Hydroxy-5-Methylisoxazole-4-Propionic Acid Receptors. Biol. Psychiatry.

[67] Moghaddam B., Adams B., Verma A., Daly D. (1997). Activation of Glutamatergic Neurotransmission by Ketamine: A Novel Step in the Pathway from NMDA Receptor Blockade to Dopaminergic and Cognitive Disruptions Associated with the Prefrontal Cortex. J. Neurosci..

[68] Lepack A.E., Fuchikami M., Dwyer J.M., Banasr M., Duman R.S. (2015). BDNF Release Is Required for the Behavioral Actions of Ketamine. Int. J. Neuropsychopharmacol..

[69] Jourdi H., Hsu Y.-T., Zhou M., Qin Q., Bi X., Baudry M. (2009). Positive AMPA Receptor Modulation Rapidly Stimulates BDNF Release and Increases Dendritic mRNA Translation. J. Neurosci..

[70] Miller O.H., Moran J.T., Hall B.J. (2016). Two Cellular Hypotheses Explaining the Initiation of Ketamine’s Antidepressant Actions: Direct Inhibition and Disinhibition. Neuropharmacology.

[71] Nosyreva E., Szabla K., Autry A.E., Ryazanov A.G., Monteggia L.M., Kavalali E.T. (2013). Acute Suppression of Spontaneous Neurotransmission Drives Synaptic Potentiation. J. Neurosci..

[72] Lin P.-Y., Ma Z.Z., Mahgoub M., Kavalali E.T., Monteggia L.M. (2021). A Synaptic Locus for TrkB Signaling Underlying Ketamine Rapid Antidepressant Action. Cell Rep..

[73] Lepack A.E., Bang E., Lee B., Dwyer J.M., Duman R.S. (2016). Fast-Acting Antidepressants Rapidly Stimulate ERK Signaling and BDNF Release in Primary Neuronal Cultures. Neuropharmacology.

[74] Liu R.-J., Lee F.S., Li X.-Y., Bambico F., Duman R.S., Aghajanian G.K. (2012). Brain-Derived Neurotrophic Factor Val66Met Allele Impairs Basal and Ketamine-Stimulated Synaptogenesis in Prefrontal Cortex. Biol. Psychiatry.

[75] Deyama S., Bang E., Kato T., Li X.-Y., Duman R.S. (2019). Neurotrophic and Antidepressant Actions of Brain-Derived Neurotrophic Factor Require Vascular Endothelial Growth Factor. Biol. Psychiatry.

[76] Kato T., Fogaça M.V., Deyama S., Li X.-Y., Fukumoto K., Duman R.S. (2018). BDNF Release and Signaling Are Required for the Antidepressant Actions of GLYX-13. Mol. Psychiatry.

[77] Kim J.-W., Suzuki K., Kavalali E.T., Monteggia L.M. (2023). Bridging Rapid and Sustained Antidepressant Effects of Ketamine. Trends Mol. Med..

[78] Kim J.-W., Autry A.E., Na E.S., Adachi M., Björkholm C., Kavalali E.T., Monteggia L.M. (2021). Sustained Effects of Rapidly Acting Antidepressants Require BDNF-Dependent MeCP2 Phosphorylation. Nat. Neurosci..

[79] Cohen S., Gabel H.W., Hemberg M., Hutchinson A.N., Sadacca L.A., Ebert D.H., Harmin D.A., Greenberg R.S., Verdine V.K., Zhou Z. (2011). Genome-Wide Activity-Dependent MeCP2 Phosphorylation Regulates Nervous System Development and Function. Neuron.

[80] Hutchinson A.N., Deng J.V., Cohen S., West A.E. (2012). Phosphorylation of MeCP2 at Ser421 Contributes to Chronic Antidepressant Action. J. Neurosci..

[81] Yao W., Cao Q., Luo S., He L., Yang C., Chen J., Qi Q., Hashimoto K., Zhang J. (2022). Microglial ERK-NRBP1-CREB-BDNF Signaling in Sustained Antidepressant Actions of (R)-Ketamine. Mol. Psychiatry.

[82] Wang Y.-L., Han Q.-Q., Gong W.-Q., Pan D.-H., Wang L.-Z., Hu W., Yang M., Li B., Yu J., Liu Q. (2018). Microglial Activation Mediates Chronic Mild Stress-Induced Depressive- and Anxiety-like Behavior in Adult Rats. J. Neuroinflamm..

[83] Mandal G., Kirkpatrick M., Alboni S., Mariani N., Pariante C.M., Borsini A. (2024). Ketamine Prevents Inflammation-Induced Reduction of Human Hippocampal Neurogenesis via Inhibiting the Production of Neurotoxic Metabolites of the Kynurenine Pathway. Int. J. Neuropsychopharmacol..

[84] Onodera J., Nagata H., Nakashima A., Ikegaya Y., Koyama R. (2021). Neuronal Brain-derived Neurotrophic Factor Manipulates Microglial Dynamics. Glia.

[85] Djalali S., Höltje M., Große G., Rothe T., Stroh T., Große J., Deng D.R., Hellweg R., Grantyn R., Hörtnagl H. (2005). Effects of Brain-Derived Neurotrophic Factor (BDNF) on Glial Cells and Serotonergic Neurones During Development. J. Neurochem..

[86] Abdallah C.G., Averill L.A., Gueorguieva R., Goktas S., Purohit P., Ranganathan M., Sherif M., Ahn K.-H., D’Souza D.C., Formica R. (2020). Modulation of the Antidepressant Effects of Ketamine by the mTORC1 Inhibitor Rapamycin. Neuropsychopharmacology.

[87] Averill L.A., Averill C.L., Gueorguieva R., Fouda S., Sherif M., Ahn K.-H., Ranganathan M., D’Souza D.C., Southwick S.M., Sanacora G. (2022). mTORC1 Inhibitor Effects on Rapid Ketamine-Induced Reductions in Suicidal Ideation in Patients with Treatment-Resistant Depression. J. Affect. Disord..

[88] Tsokas P., Blitzer R.D., Sajikumar S. (2015). mTOR and the Regulation of Translational Capacity in Late Forms of Synaptic Plasticity. Synaptic Tagging and Capture.

[89] Izumi Y., Zorumski C.F. (2014). Metaplastic Effects of Subanesthetic Ketamine on CA1 Hippocampal Function. Neuropharmacology.

[90] Cavalleri L., Merlo Pich E., Millan M.J., Chiamulera C., Kunath T., Spano P.F., Collo G. (2018). Ketamine Enhances Structural Plasticity in Mouse Mesencephalic and Human iPSC-Derived Dopaminergic Neurons via AMPAR-Driven BDNF and mTOR Signaling. Mol. Psychiatry.

[91] Maiworm M. (2024). The Relevance of BDNF for Neuroprotection and Neuroplasticity in Multiple Sclerosis. Front. Neurol..

[92] Kang M.J.Y., Hawken E., Vazquez G.H. (2022). The Mechanisms Behind Rapid Antidepressant Effects of Ketamine: A Systematic Review with a Focus on Molecular Neuroplasticity. Front. Psychiatry.

[93] Mohammad Shehata I., Masood W., Nemr N., Anderson A., Bhusal K., Edinoff A.N., Cornett E.M., Kaye A.M., Kaye A.D. (2022). The Possible Application of Ketamine in the Treatment of Depression in Alzheimer’s Disease. Neurol. Int..

[94] Haile C.N., Murrough J.W., Iosifescu D.V., Chang L.C., Al Jurdi R.K., Foulkes A., Iqbal S., Mahoney J.J., De La Garza R., Charney D.S. (2014). Plasma Brain Derived Neurotrophic Factor (BDNF) and Response to Ketamine in Treatment-Resistant Depression. Int. J. Neuropsychopharm..

[95] Duncan W.C., Sarasso S., Ferrarelli F., Selter J., Riedner B.A., Hejazi N.S., Yuan P., Brutsche N., Manji H.K., Tononi G. (2013). Concomitant BDNF and Sleep Slow Wave Changes Indicate Ketamine-Induced Plasticity in Major Depressive Disorder. Int. J. Neuropsychopharmacol..

[96] Woelfer M., Li M., Colic L., Liebe T., Di X., Biswal B., Murrough J., Lessmann V., Brigadski T., Walter M. (2020). Ketamine-Induced Changes in Plasma Brain-Derived Neurotrophic Factor (BDNF) Levels Are Associated with the Resting-State Functional Connectivity of the Prefrontal Cortex. World J. Biol. Psychiatry.

[97] Machado-Vieira R., Yuan P., Brutsche N., DiazGranados N., Luckenbaugh D., Manji H.K., Zarate C.A. (2009). Brain-Derived Neurotrophic Factor and Initial Antidepressant Response to an *N*-Methyl-D-Aspartate Antagonist. J. Clin. Psychiatry.

[98] Medeiros G.C., Greenstein D., Kadriu B., Yuan P., Park L.T., Gould T.D., Zarate C.A. (2021). Treatment of Depression with Ketamine Does Not Change Plasma Levels of Brain-Derived Neurotrophic Factor or Vascular Endothelial Growth Factor. J. Affect. Disord..

[99] Wang J., Wang Y., Xu X., Peng S., Xu F., Liu P. (2020). Use of Various Doses of S-Ketamine in Treatment of Depression and Pain in Cervical Carcinoma Patients with Mild/Moderate Depression After Laparoscopic Total Hysterectomy. Med. Sci. Monit..

[100] Zheng W., Gu L., Zhou Y., Wang C., Lan X., Zhang B., Li Z., Ning Y. (2023). Baseline Plasma BDNF Levelsare Associated with Antianhedonic Effects ofRepeated-Dose Intravenous Ketamine in Major Depressive Disorder. Curr. Neuropharmacol..

[101] Wang M., Zhang B., Zhou Y., Wang C., Zheng W., Liu W., Zhan Y., Lan X., Ning Y. (2021). Sleep Improvement Is Associated with the Antidepressant Efficacy of Repeated-Dose Ketamine and Serum BDNF Levels: A Post-Hoc Analysis. Pharmacol. Rep..

[102] Grunebaum M.F., Ellis S.P., Keilp J.G., Moitra V.K., Cooper T.B., Marver J.E., Burke A.K., Milak M.S., Sublette M.E., Oquendo M.A. (2017). Ketamine Versus Midazolam in Bipolar Depression with Suicidal Thoughts: A Pilot Midazolam-Controlled Randomized Clinical Trial. Bipolar Disord..

[103] Li C., Cai Q., Su Z., Chen Z., Cao J., Xu F. (2023). Could Peripheral 5-HT Level Be Used as a Biomarker for Depression Diagnosis and Treatment? A Narrative Minireview. Front. Pharmacol..

[104] Jiang Q., Qi Y., Zhou M., Dong Y., Zheng W., Zhu L., Li Y., Zhou H., Wang L. (2024). Effect of Esketamine on Serum Neurotransmitters in Patients with Postpartum Depression: A Randomized Controlled Trial. BMC Anesthesiol..

[105] Luo T., Deng Z., Ren Q., Mu F., Zhang Y., Wang H. (2024). Effects of Esketamine on Postoperative Negative Emotions and Early Cognitive Disorders in Patients Undergoing Non-Cardiac Thoracic Surgery: A Randomized Controlled Trial. J. Clin. Anesth..

[106] Klein A.B., Williamson R., Santini M.A., Clemmensen C., Ettrup A., Rios M., Knudsen G.M., Aznar S. (2011). Blood BDNF Concentrations Reflect Brain-Tissue BDNF Levels across Species. Int. J. Neuropsychopharm..

[107] Karege F., Schwald M., Cisse M. (2002). Postnatal Developmental Profile of Brain-Derived Neurotrophic Factor in Rat Brain and Platelets. Neurosci. Lett..

[108] Sartorius A., Hellweg R., Litzke J., Vogt M., Dormann C., Vollmayr B., Danker-Hopfe H., Gass P. (2009). Correlations and Discrepancies Between Serum and Brain Tissue Levels of Neurotrophins after Electroconvulsive Treatment in Rats. Pharmacopsychiatry.

[109] Elfving B., Plougmann P.H., Müller H.K., Mathé A.A., Rosenberg R., Wegener G. (2010). Inverse Correlation of Brain and Blood BDNF Levels in a Genetic Rat Model of Depression. Int. J. Neuropsychopharm..

[110] Pan Z., Park C., Brietzke E., Zuckerman H., Rong C., Mansur R.B., Fus D., Subramaniapillai M., Lee Y., McIntyre R.S. (2019). Cognitive Impairment in Major Depressive Disorder. CNS Spectr..

[111] Eisch A.J., Bolaños C.A., De Wit J., Simonak R.D., Pudiak C.M., Barrot M., Verhaagen J., Nestler E.J. (2003). Brain-Derived Neurotrophic Factor in the Ventral Midbrain–Nucleus Accumbens Pathway: A Role in Depression. Biol. Psychiatry.

